# Computed tomography-derived myocardial extracellular volume: an early biomarker of cardiotoxicity in esophageal cancer patients undergoing radiation therapy

**DOI:** 10.1186/s13244-020-00922-2

**Published:** 2020-11-23

**Authors:** Davide Capra, Caterina Beatrice Monti, Alberto Gianluigi Luporini, Fabrizio Lombardi, Calogero Gumina, Andrea Sironi, Emanuele Luigi Giuseppe Asti, Luigi Bonavina, Francesco Secchi, Francesco Sardanelli

**Affiliations:** 1 Department of Biomedical Sciences for Health, Università Degli Studi Di Milano, Via Mangiagalli 31, 20133 Milano, Italy; 2 Unit of Medical Oncology, IRCCS Policlinico San Donato, Via Morandi 30, 20097 San Donato Milanese, Italy; 3 Unit of Radiation Oncology, IRCCS Policlinico San Donato, Via Morandi 30, 20097 San Donato Milanese, Italy; 4 Division of General and Foregut Surgery, IRCCS Policlinico San Donato, Via Morandi 30, 20097 San Donato Milanese, Italy; 5 Unit of Radiology, IRCCS Policlinico San Donato, Via Morandi 30, 20097 San Donato Milanese, Italy

**Keywords:** Esophageal neoplasms, Tomography X-ray computed, Cardiotoxicity, Radiotherapy, Extracellular space

## Abstract

**Objectives:**

We aimed to assess extracellular volume (ECV) through non-gated, contrast-enhanced computed tomography (CT) before and after radiation therapy (RT) in patients with esophageal cancer (EC).

**Materials and methods:**

EC patients who had undergone CT before and after RT were retrospectively assessed. Patients with preexisting cardiovascular disease or with heavily artifacted CT were excluded. ECV was calculated using density values for the myocardial septum and blood pool. Data were reported as mean and standard deviation or median and interquartile range according to their distribution; *t* test or Wilcoxon and Pearson r or Spearman ρ were subsequently used.

**Results:**

Twenty-one patients with stage ≥ IB EC, aged 64 ± 18 years, were included. Mean and maximum RT doses were 21.2 Gy (16.9–24.1) and 42.5 Gy (41.8–49.2), respectively. At baseline (*n* = 21), hematocrit was 39% ± 4%, ECV 27.9% ± 3.5%; 35 days (30–38) after RT (*n* = 20), hematocrit was 36% ± 4%, lower than at baseline (*p* = 0.002), ECV 30.3% ± 8.3%, higher than at baseline (*p* = 0.081); at follow-up 420 days (244–624) after RT (*n* = 13), hematocrit was 36% ± 5%, lower than at baseline (*p* = 0.030), ECV 31.4% ± 4.5%, higher than at baseline (*p* = 0.011). No patients showed signs of overt cardiotoxicity. ECV early after RT was moderately positively correlated with maximum RT dose (ρ = 0.50, *p* = 0.036).

**Conclusions:**

In EC patients, CT-derived myocardial ECV was increased after RT and may thus appear as a potential early biomarker of cardiotoxicity.

## Key points

- Non-gated CT-derived ECV increased after radiotherapy in esophageal cancer patients.
- CT-derived ECV may help detect early changes in myocardial tissue from cardiotoxicity.
- Further studies need to define the role of CT-derived ECV in cardiotoxicity.

## Introduction

Esophageal cancer (EC), with 572,000 estimated new cases [1], and 509,000 estimated deaths in 2018 [2], ranks seventh in terms of incidence and sixth in terms of mortality worldwide [3]. In locally advanced disease, the combination of chemotherapy and radiotherapy provides beneficial effects to patients, increasing the overall survival [4]. Nevertheless, this treatment scheme is burdened by the risk of side effects, cardiotoxicity being one of the major concerns as it poses a serious threat to long-term survival [5]. In particular, high doses of radiation therapy appear to play the biggest role in cardiotoxicity. Radiation damage to the heart is characterized by acute and chronic modifications in cardiac tissue, ultimately leading to cardiac dysfunction due to myocardial fibrosis [6]. As recently described by Xu et al. [7], radiation dose to the heart is an independent predictor of overall survival for EC patients, and a cardiac volume receiving 30 Gy > 45% is associated with worse survival.

Early detection of asymptomatic cardiac damage during treatment could drive subsequent clinical decisions, including starting cardioprotective therapy, evaluating different treatment protocols, or implementing a closer follow-up strategy [8]. Recent statements by the European Society of Cardiology highlight the need for greater acknowledgement and serial monitoring of heart failure in cancer survivors [8, 9]. In fact, novel evidence showed that cancer patients who develop subclinical left ventricular dysfunction or heart failure during or after therapy could benefit from treatment with angiotensin-converting enzyme inhibitors, angiotensin II receptor blockers, or beta blockers [8]. However, the timely detection of cardiotoxicity is hindered by the vast functional reserve of the myocardium, and overt functional loss is only evident from a decrease in ejection fraction after a substantial amount of damage has occurred [10]. Thus, more sensitive diagnostic tools are needed to assess the earlier stages of cardiac damage.

In this light, extracellular volume (ECV) is emerging as a sensitive biomarker of myocardial fibrosis [11]. The assessment of ECV allows the estimation of the myocardial volume fraction that is not composed by myocytes. ECV measured with dedicated magnetic resonance (MR) pulsed sequences has demonstrated a strong, positive correlation with histological collagen volume fraction [12]. Calculating ECV from dedicated computed tomography (CT) scans was proven to be feasible by recent works, which highlighted a strong, positive correlation between CT-derived ECV and MR-derived ECV or histological findings [13]. An increase in ECV thus may offer valuable information for heart failure or cardiac-related death [14].

For staging and follow-up purposes, EC patients currently undergo non-cardiac dedicated and thus not electrocardiographically gated, contrast-enhanced CT scans of the chest, according to current guidelines [4]. These scans, however, allow a sufficiently good visualization of the heart both on unenhanced and contrast-enhanced scans, allowing ECV estimation, potentially providing additional information concerning patients’ cardiac condition.

Hence, our study aimed to assess ECV through non-gated, contrast-enhanced CT in EC patients and to ascertain whether an increase in CT-derived ECV after radiation therapy can be observed.

## Materials and methods

### Ethics committee

This study was approved by the local ethics committee (Ethics Committee of San Raffaele Clinical Research Hospital; protocol code “CardioRetro,” number 122/int/2017; approved on September 14, 2017, and amended July 18, 2019). Specific informed consent was waived due to the retrospective nature of this study. This study was partially supported by Ricerca Corrente funding from Italian Ministry of Health to IRCCS Policlinico San Donato.

### Study population

Patients with a history of thoracic EC, who had one contrast-enhanced CT examinations before radiation therapy and one or two contrast-enhanced CT examinations after radiation therapy performed at our institution between November 2011 and February 2019, were retrieved from the picture archiving and communication system of our institution. Exclusion criteria were: (1) not having undergone radiation therapy; (2) cardiac comorbidities such as arrhythmias, coronary artery disease, or any cardiac pathology that may imply underlining fibrosis represented by low ejection fraction (≤ 45%), to avoid any possible confounding factor altering the attenuation measurement of the myocardial tissue; (3) no hematocrit values measured between four weeks prior and four weeks after the CT examination needed for enrolment.

### Esophageal cancer treatment

All patients received radiation therapy, and some patients also underwent chemotherapy. Concerning radiation therapy, most patients were treated with volumetric modulated arc therapy, receiving a median dose of 41.4 Gy in 23 fractions (1.8 Gy per fraction) [15]. Chemotherapy protocols included combinations of either cisplatin and 5-fluorouracil or carboplatin and taxanes in variable doses, according to patient fitness and body surface area.

### Image acquisition

Patients were imaged using a 64-row CT scan (Somatom Definition, Siemens, Healthineers, Erlangen, Germany) with 120 kVp, tube current ranging from 157 to 236 mAs depending on automatic exposure control system (CARE Dose 4D, Siemens, Healthineers, Erlangen, Germany), 0.5 s of rotation speed, pitch 1, B30f medium smooth for kernel recon technique and abdomen window, or a 16-row CT scan (Emotion 16, Siemens, Healthineers, Erlangen, Germany) with 130 kVp, tube current ranging from 55 to 234 mAs depending on automatic exposure control system (CARE Dose 4D, Siemens, Healthineers, Erlangen, Germany), 0.5 s of rotation speed, pitch 1, B30f medium smooth for kernel recon technique. Iopamidol (Iopamiro 370; 370 mg I/mL; Bracco Imaging, Milan, Italy) was administered based on patient total body weight. The contrast agent was administered intravenously through a 20-gauge needle using an automatic power injector (EmpowerCTA Contrast Injection System, Bracco Imaging, Milan, Italy) at the rate of 3 mL/s, followed by 50 mL of saline solution at the same rate. Scan delay was determined using an automated triggering hardware and a dedicated software (Bolus Tracking, Siemens, Healthineers, Erlangen, Germany). Specifically, low-dose monitor images were obtained in a single axial slice of the aorta after contrast agent injection. Approximately 80 s after the descending aorta reached 100 Hounsfield units, a portal venous phase scan was acquired.

### Image analysis

**Fig. 1:**
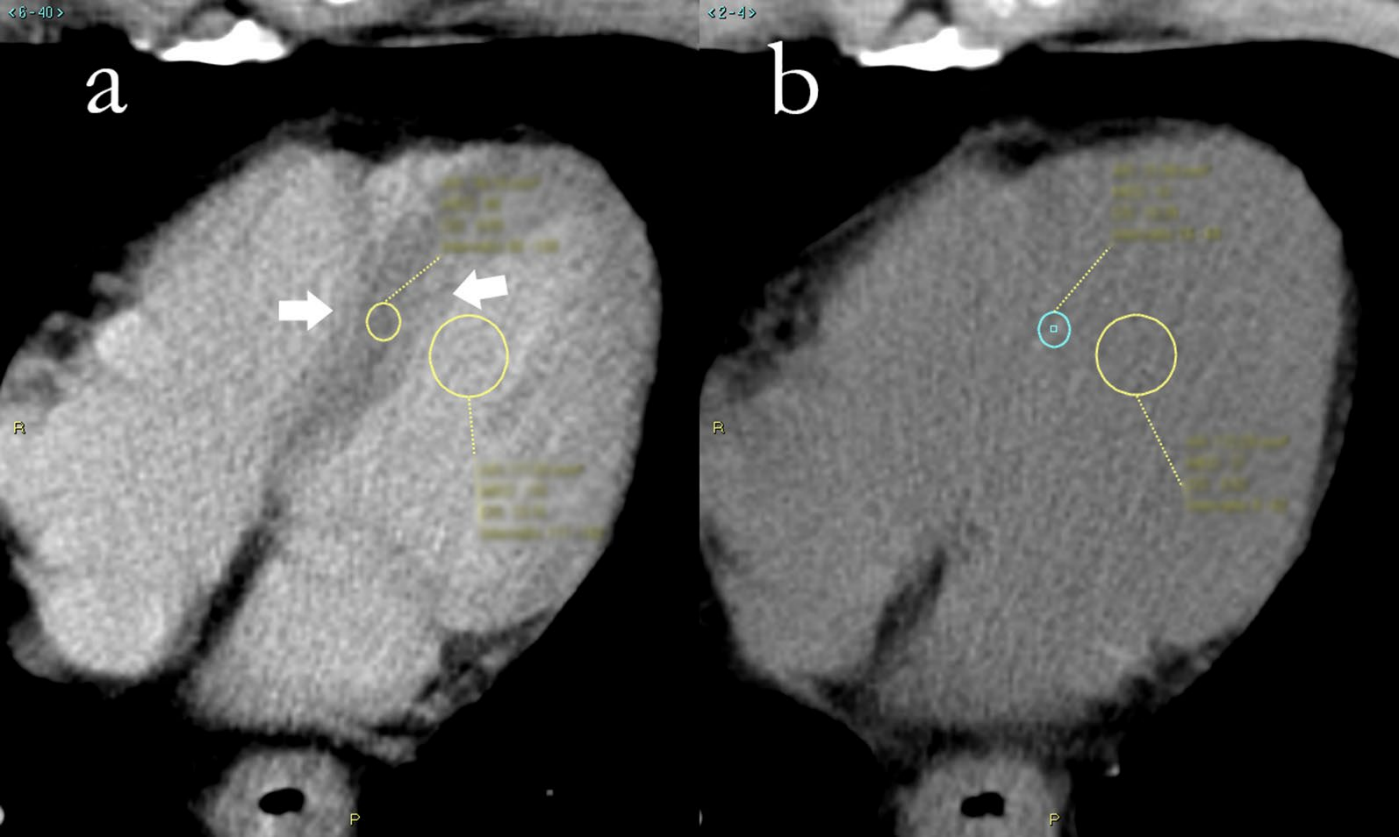
Region of interest placement for extracellular volume calculation in computed tomography of a 65-year-old male patient with adenocarcinoma of lower esophagus. **a** Contrast-enhanced scan, where papillary muscles are visible and thus avoided. The septum is clearly distinguishable from the intraventricular blood pool, and a certain degree of blurring is noticeable toward the borders (white arrows). **b** Unenhanced scan. Myocardial tissue is almost unrecognizable from the intraventricular blood pool, and regions of interest are placed in roughly the same position of **a** and then adjusted following local attenuation measurement

All images were reviewed by two readers with one and two years of experience in cardiac CT, respectively. First, the reader chose the axial slice which best allowed the visualization of the left ventricle. A round region of interest (ROI) with a minimum area of 25 mm^2^ was placed in the ventricular septum, to obtain its attenuation measurement. Since these CT scans were not electrocardiographically gated, the effects of cardiac movement were taken into consideration when placing the ROIs in the middle of the septum, excluding the eternal portions closer to the intraventricular right and left blood pool, prone to be more blurred as a consequence of cardiac movement. Similarly, a second ROI, with a minimum area of 130 mm^2^, was placed in the intraventricular left blood pool on the same image, avoiding papillary muscles, as shown in Fig. 1. This was first done on the contrast-enhanced scans, as myocardium and blood pool were clearly recognizable, and then, ROIs were placed in the same positions on unenhanced scans.

ECV was calculated using the formula as proposed by Bandula et al. [13]:

$$ECV = \left( {1 - Hematocrit} \right) \times \left[ {\frac{{HU_{{myo_{post} }} - HU_{{myo_{pre} }} }}{{HU_{{blood_{post} }} - HU_{{blood_{pre} }} }}} \right]$$

where *myo* = myocardium; *pre* = pre-contrast; *post* = post-contrast.

### Statistical analysis

Shapiro–Wilk tests were conducted to assess data distribution. Normally distributed data were reported as mean ± standard deviation. Non-normally distributed data were reported as median and interquartile range (IQR). Measurements were compared using two-sided Student’s t test for paired data for normal distributions or Wilcoxon test for non-normal distributions. Pearson and Spearman correlation tests were used according to data distribution. Correlation coefficients were interpreted according to Evans [16]. Bland–Altman analysis was conducted to assess intra- and inter-reader reproducibility, which was reported as bias, coefficient of repeatability and reproducibility index, namely the complement to one of the ratio between bias and mean measure. We defined a reproducibility index as the complement to 1 of the ratio between the double of the coefficient of repeatability and the mean of all measures. Statistical analysis was performed with R v3.5.3. *P* values < 0.05 were considered as significant [17].

## Results

### Study population

**Fig. 2:**
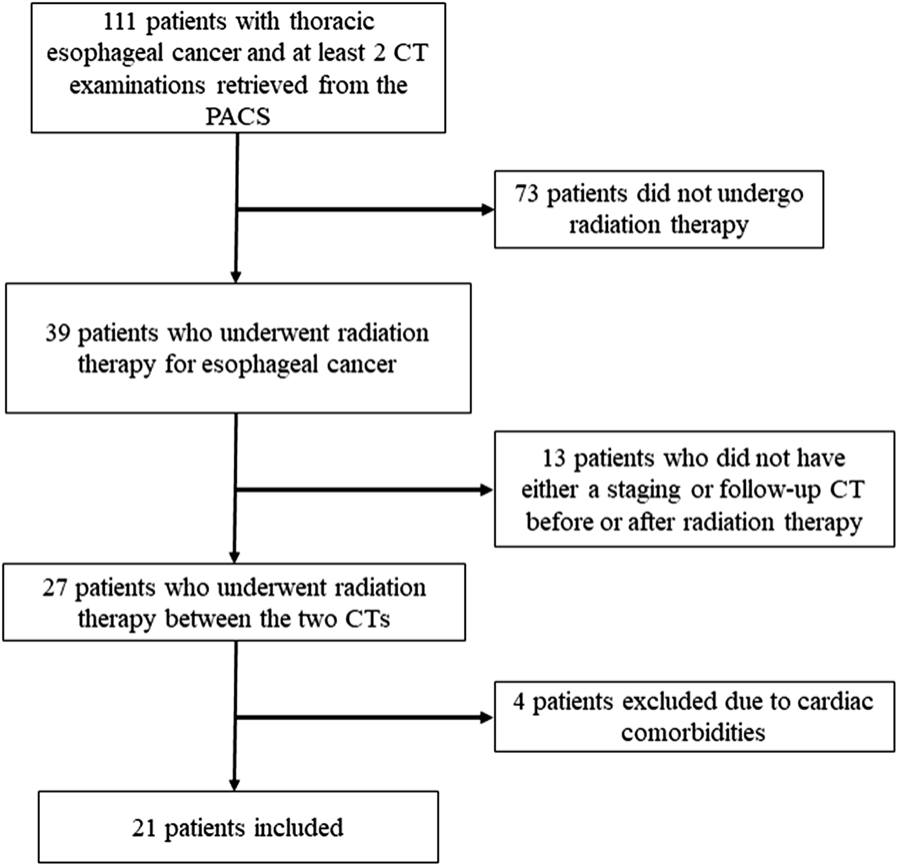
Inclusion flowchart. PACS: picture archiving and communication system; CT: computed tomography

One hundred and eleven patients were initially identified for our study. Seventy-three patients had not undergone radiation therapy and were then excluded. Thirteen more patients were excluded because they did not have either a staging or a follow-up CT examination either before or after radiation therapy. Eventually, four patients were excluded due to preexisting cardiac comorbidities (ejection fraction ≤ 45%, n = 4). Hence, 21 patients were included in the study. Patients selection is illustrated in Fig. 2.

Four patients were females and 17 males. The age at baseline was 64 ± 18 years. Mean body mass index was 23.6 ± 3.7; 10/21 patients (48%) were smokers, 7/21 (33%) were habitual drinkers, and 2/21 (10%) suffered from type 2 diabetes. Tumor histological type and location are reported in Table 1. All patients had stage IB or higher disease. Ejection fraction was deemed above 45% in all patients and remained so at all time points. No pericardial effusion was observed [18].

**Table 1:** Patients characteristics

Number of patients	21
Age at diagnosis (years)	64 ± 18
Females (n, %)	4 (19)
Body mass index (kg/m^2^)	23.7 ± 3.7
Smokers (n, %)	10 (48)
Alcohol (n, %)	7 (33)
Diabetes (n, %)	2 (10)
Location of tumor (n, %)	
Upper thoracic esophagus	1 (5)
Middle thoracic esophagus	5 (24)
Lower thoracic esophagus	14 (66)
Unknown	1 (5)
Tumor type (n, %)	
Squamous cell carcinoma	13 (62)
Adenocarcinoma	6 (28)
Unknown	2 (10)

Continuous data are presented as mean ± standard deviation

### Radiation therapy

All but one patient received RapidArc radiation therapy, with a median dose of 41.4 Gy (range 32.4–61.6 Gy). Median heart dose was 21.2 Gy (IQR 16.9–24.1 Gy). Median maximum heart dose was 42.5 Gy (IQR 41.8–49.2 Gy). The median percentage of cardiac volume receiving a radiation dose of 20 Gy (V20) was 46.6% (IQR 25.2–61.0%), V30 was 16.2% (IQR 6.9–24.0%), and V40 was 1.8% (IQR 0.4–9.6%).

### Chemotherapy

Fifteen patients received chemotherapy with dosages adjusted according to patients’ fitness and body surface area. Eleven patients received a combination of carboplatin (doses ranging from 160 to 230 mg/m^2^) and paclitaxel (from 70 to 125 mg/m^2^) for 4–10 cycles. Four patients received a combination of cisplatin (from 25 to 75 mg/m^2^) and 5-fluorouracil (from 685 to 750 mg/m^2^) for 4–5 cycles. One patient received both schemes, one patient received oxaliplatin 100 mg and 5-fluorouracil 685 mg/m^2^ in addition to the carboplatin and paclitaxel scheme, and one patient received docetaxel 75 mg/m^2^ in addition to cisplatin and 5-fluorouracil scheme.

### Extracellular volume variations with radiation therapy

Mean hematocrit at baseline was 39% ± 4%. Before radiation therapy, CT-derived ECV was 27.9% ± 3.5%.

Twenty-one patients underwent CT early after radiation therapy, with a median interval from the end of radiation therapy of 35 days (IQR 30–38 days). Hematocrit was 36% ± 4%, significantly lower than pre-treatment values (*p* = 0.002). Average ECV was 30.3% ± 8.3%, with an increase versus prior to radiation therapy, although not significant (*p* = 0.081).

Thirteen patients had an available follow-up, with a median interval from the end of radiation therapy of 420 days (IQR 244–624 days). Hematocrit was 36% ± 5%, significantly lower than pre-treatment values (*p* = 0.030). At follow-up, we found a mean value of ECV of 31.4% ± 4.5%, significantly higher than baseline (*p* = 0.011). No patients showed signs of cardiotoxicity from baseline to follow-up.

**Fig. 3:**
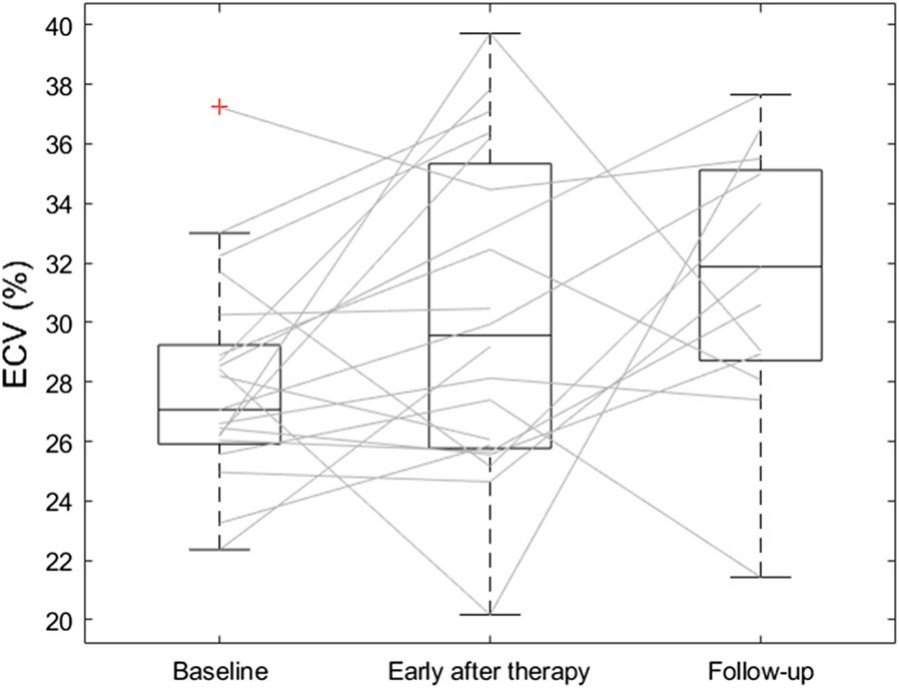
Paired boxplot of extracellular volume (ECV) measurements at different time points: baseline before radiation therapy, early after radiation therapy, and at follow-up. Lines mark ECV evolution for each patient

A boxplot depicting ECV at different time points is reported in Fig. 3. Data regarding hematocrit and ECV are reported in Table 2.

**Table 2:** Hematocrit and extracellular volume (ECV) at different time points: baseline, early after radiation therapy, and follow-up

	Baseline	Early after radiation therapy	Follow-up
Hematocrit (%)	39 ± 4	36 ± 4	36 ± 5
ECV (%)	27.9 ± 3.5	30.3 ± 5.4	31.4 ± 4.5

Data are presented as mean ± standard deviation

Concerning correlations between ECV and radiation therapy data, ECV early after radiation therapy exhibited a moderate positive correlation with the maximum myocardial dose (ρ = 0.50, *p* = 0.036) and trended toward a moderate positive correlation with V40 (ρ = 0.46, *p* = 0.058). None of the other parameters showed significant correlations with ECV early after radiation therapy (*p* ≥ 0.467). ECV at follow-up did not correlate with any radiation therapy parameter (*p* ≥ 0.196).

### Reproducibility

Bland–Altman analysis for inter-reader reproducibility showed a bias of − 1.26% and a coefficient of repeatability of 7.25% with a reproducibility index of 76%.

## Discussion

Our study assessed changes in myocardial ECV after radiation therapy on CT scans in a population of 21 patients with thoracic EC. We compared baseline CT-derived ECV to post-treatment and follow-up ECV, finding a trend toward an increased ECV at 1 month after therapy that was confirmed by a significant ECV increase versus the baseline value at a median follow-up of around 14 months.

The baseline myocardial ECV found in this study is compatible with that reported by Takagi et al. [19] using cardiac MR T1 mapping, albeit with a trend toward higher values. Considering that our patients did not have any history or signs of cardiac disease, this plays in favor of the hypothesis that non-gated, contrast-enhanced CT-derived ECV estimates, obtained in the portal phase, are consistent with those obtained by cardiac MR at equilibrium. Moreover, a higher CT-derived ECV value compared to that MR derived may be expected, as this difference is already known from previous studies [13]. Our results concerning baseline ECV were also compatible with those reported by Nacif et al. [20] in healthy subjects, suggesting the possibility of estimating ECV from non-gated CT, which means not being obligated to use electrocardiographically gated cardiac CT for this clinical aim, possibly sparing further radiation exposition to patients [21].

Patients were treated with radiation therapy according to current guidelines [15]. Heart irradiation may cause microvascular damage, leading to an early phase of inflammation followed by the onset of fibrosis, both processes causing an increase in ECV. However, the expected increase in ECV was not statistically significant early after radiation therapy. This could be explained by the high heterogeneity of response to cardiac damage stemming from radiation therapy in different patients. In fact, ECV estimates obtained early after radiation therapy showed a wide standard deviation, and we observed different responses from patient to patient. Some patients displayed a rise in ECV, while others maintained the same ECV or even showed a decrease in ECV. This heterogeneity might be related to individual sensitivity to acute radiation damage and to differences in radiation dose to the heart. This hypothesis is also supported by the modest correlation between maximum myocardial dose or V40 and ECV values early after radiation therapy. Moreover, it has been reported that radiation-related myocardial injury is mostly a late occurring event [22]. Thus, substantial changes in myocardial tissue are expected to appear in a time span longer than a month. In fact, we found a significant ECV increase at a median time of 420 days, most likely due to the delayed onset of myocardial fibrosis. As no patients displayed signs attributable to cardiac toxicity, this finding suggests CT-derived ECV as a biomarker of radiation therapy-induced cardiac damage. Individual variability of baseline and post-treatment ECT estimates deserves careful analysis in future prospective studies, also assessing the role of different chemotherapy regimens.

EC patients face the threat of cardiac pathology, arising as a consequence of combined chemoradiotherapy [23], causing myocardial fibrosis and subsequent heart failure. With the improvements in survival rates [24], this is likely to become a primary concern. In this context, MR-derived ECV can be proposed as a good biomarker for myocardial fibrosis, allowing its early detection, and it has been validated against histological findings [19]. However, cardiac MR is not part of the routine workflow of EC patients, whereas thoracic contrast-enhanced, non-gated CT is. Hence, deriving ECV values from routine CT would allow a prompt detection of cardiac fibrosis, without adding further investigations to patients. This is a non-negligible advantage of our study and opens to the perspective of a clinical routine use of this parameter in the emerging world of cardio-oncology. Furthermore, ECV values in our study showed substantial reproducibility, prompting the reliability of this measure.

A few notable limitations must be mentioned. First, ECV is usually assessed at equilibrium that would be reached at least 5 min after the injection of contrast [20]. Although this might be ideal, most oncologic follow-up CT protocols do not include delayed phase scans after the portal venous phase, in order to spare radiation exposure to the patient. A previous work [25] showed good correlations between myocardial ECV values obtained at 1, 3, and 7 min after contrast injection in breast cancer patients who underwent thoracic CT; hence, we can speculate that the portal venous phase we considered in the current work (approximately 80 s after contrast injection) allowed to measure values of relative enhancement readable as CT-derived ECV values. Secondly, most of our patients underwent combined chemoradiotherapy, making it impossible to discern the individual impact of either radiation or drug therapies. Nonetheless, it seems unlikely for the chemotherapeutic drugs used in the present study (5-fluorouracil, cisplatin, carboplatin, and paclitaxel) to impact the ECV at follow-up, as they tend to be associated with acute cardiotoxicity, that none of the subjects of the study experienced, but not with long-term cardiac damage [26, 27]. Third, two different CT scanners were used (16 or 64 slices), with slightly different tube voltages (130 kVp and 120 kVp, respectively) and automatic exposure (157–236 mAs and 55–234 mAs, respectively). Nevertheless, Hounsfield units are standardized; therefore, the only practical issue should have been the cardiac movement, which was accounted for by ROI placement in the myocardial septum. Furthermore, we did not assess the impact of image noise or beam-hardening artifacts, which could impact the accuracy of ECV measures [28]. Future studies assessing the impact of noise and beam-hardening artifacts on non-gated CT-derived ECV values are needed, and, in this aspect, different iterative reconstruction algorithms showed promising results in improving image quality [29] and accuracy of ECV measures [28]. Finally, given the retrospective and monocentric nature of the study we were able to include only a small number of patients, reducing the statistical power of the study. Despite these limitations, our ECV values showed to be compatible to that found in other studies [19, 20], suggesting the reliability of our method.

In conclusion, our study showed an increase in ECV obtained from non-gated CT after treatment in EC patients after radiation therapy in the absence of overt cardiac pathology, suggesting the ability of this biomarker to detect early changes in myocardial tissue in this setting. Further perspective, multicenter studies are warranted to define a normal range for non-cardiac CT-derived ECV and to establish its prognostic value in predicting long-term cardiac events in EC patients after radiation therapy.

## Data Availability

The datasets used and/or analyzed during the current study are available from the corresponding author on reasonable request.

## Abbreviations

CT: Computed tomography
EC: Esophageal cancer
ECV: Extracellular volume
IQR: Interquartile range
MR: Magnetic resonance
ROI: Region of interest
